# Community-informed trial design and implementation to optimize recruitment and retention in low-income settings: lessons from rheumatic heart disease trials in Uganda

**DOI:** 10.3389/fcvm.2026.1851733

**Published:** 2026-07-13

**Authors:** Juliet Alepere, Susan Akullo, Pamela Okwir Apio, Brenda Atim, Andrea Beaton, Anneke Grobler, Samalie Kitooleko, Haddy Nalubwama, Emma Ndagire, Emmy Okello, Isaac Otim Omara, Jafes Pulle, Joselyn Rwebembera, Craig Sable, Amy Scheel, Alison Spaziani, Andrew Steer, Rachel Sarnacki

**Affiliations:** 1 RHD Research Collaborative in Uganda, Uganda Heart Institute, Kampala, Uganda; 2 Department of Pediatrics, School of Medicine, University of Cincinnati, Cincinnati, OH, United States; 3 Division of Cardiology, The Heart Institute, Cincinnati Children’s Medical Center, Cincinnati, OH, United States; 4 Murdoch Children’s Research Institute, Melbourne, VIC, Australia; 5 Department of Paediatrics, University of Melbourne, Melbourne, VIC, Australia; 6 Division of Paediatric Cardiology, Department of Paediatric Cardiology, Uganda Heart Institute, Kampala, Uganda; 7 Division of Adult Cardiology, Department of Adult Cardiology, Uganda Heart Institute, Kampala, Uganda; 8 Division of Cardiology, Ochsner Children’s Hospital, New Orleans, LA, United States; 9 Xavier Ochsner College of Medicine (XOCOM), New Orleans, LA, United States; 10 Division of Cardiology, Children’s Hospital of Philadelphia, Philadelphia, PA, United States; 11 Global Health Initiative, Division of Cardiology, Children’s National Hospital, Washington, DC, United States; 12 Melbourne Children’s Global Health, Royal Children’s Hospital, Melbourne, VIC, Australia

**Keywords:** adherence, community, trial consent, engagement, low-resource, pediatric, trial retention, rheumatic heart disease

## Abstract

Research teams undertaking randomized clinical trials in low-and middle-income countries frequently encounter significant challenges, including limited infrastructure, cultural barriers to trust, and limited resources. Rheumatic heart disease (RHD) trials can be especially difficult because early stages of RHD often show no symptoms, there is low disease awareness among healthcare workers and the public, pediatric heart disease is often stigmatized, and disease management requires burdensome secondary antibiotic prophylaxis. To address these challenges, the RHD Research Collaborative in Uganda (RRCU) partnered with community members to establish a community-centered framework for trial design and implementation that incorporates group-based consent, frequent and personalized communication, and convenient, family-friendly participation structures. These components have been successfully adapted and applied across a series of RRCU studies, contributing to exceptionally high rates of consent, retention, and prophylaxis adherence. The RRCU's community-informed approach to trial design and implementation challenges assumptions about research feasibility in low-resource settings and demonstrates that high-quality, ethical pediatric research can be successfully conducted by engaging communities and integrating their values into all aspects of study design and execution.

## 1 Introduction

### 1.1 Challenges to trial design and implementation

Conducting randomized clinical trials presents persistent challenges related to community engagement, participant recruitment, and long-term retention (1). These barriers are especially challenging in low- and middle-income countries (LMIC) where limited research infrastructure, cultural factors affecting trust in medical research, shortage of trained personnel, and inadequate funding create substantial obstacles to trial conduct (2, 3). Further, recruitment and consent approaches mandated by partner institutions in high-resource settings may not directly translate to LMIC contexts, risking inadequate protection of participant autonomy and unintentionally undermining the purpose of informed consent (4).

Research challenges are particularly pronounced in the context of rheumatic heart disease (RHD), a neglected cardiovascular condition that remains endemic in LMICs. RHD, which is associated with conditions of social deprivation, affects at least 54.8 million people and causes more than 370,000 deaths each year (5, 6). As the leading acquired cardiovascular disease among children and young adults, RHD accounts for the greatest cardiovascular disease-related loss of disability-adjusted life years among 10–14 year olds (7).

RHD trial environments are highly complex due to limited awareness among both communities and healthcare providers. Added barriers include the often-asymptomatic presentation of early RHD, stigma surrounding pediatric heart disease, the complexity of this non-communicable disease, which has infectious and immunological origins, and the considerable burden of prolonged secondary antibiotic prophylaxis (8–10). Studies have documented RHD secondary prophylaxis adherence as low as 27% in LMIC settings, with patient-, family-, and health system-level barriers contributing to poor retention in longitudinal care (11).

### 1.2 A multi-stage approach to community consultation

To address barriers to conducting RHD trials in Uganda and other LMIC settings, The Rheumatic Heart Disease Research Collaborative in Uganda (RRCU) customized and implemented an iterative community-informed approach to trial design and implementation. This method engages the community early through “community engagement studios” and continues consultation throughout the trial through a Community Advisory Board. Its structured yet flexible design allows investigators to respond to community concerns and incorporate feedback into trial protocols, while still allowing for adaptation and innovation as the work evolves.

### 1.3 Success within the GOAL-family of studies

RRCU has successfully applied these approaches in Uganda's Gulu and Lira districts through the RRCU's GOAL-family of five studies, which collectively aim to define the standard of prophylactic and long-term care for children with early RHD that is detected through echocardiographic screening. The results outlined below include examples of trial design and implementation tactics created for and used across the GOAL-family of studies, with particular focus on the GOAL and GOALIE trials. The GOAL trial (NCT03346525) was designed to determine if secondary antibiotic prophylaxis prevents the progression of early RHD, and the GOALIE trial (NCT05693545) aims to determine if oral penicillin prophylaxis is non-inferior to injectable benzathine penicillin G prophylaxis in protecting children with mild RHD from progression to moderate/severe disease. Demonstrating of their impact, RRCU's multi-stage community consultation and implementation process contributed to exceptionally high consent rates (>99%), retention [>97% at trial completion (2 years)], and adherence to monthly injections (>99%) in the GOAL trial (12–14).

### 1.2 A broader application

In this experience-from-the-field commentary, we describe our community engagement methods for trial design and implementation, including the community-informed frameworks used for recruitment and retention. We also detail how these approaches have evolved over time in response to an increasingly nuanced understanding of population needs and local context. We aim to provide a practical and transferable example of a successful community-driven approach that can strengthen the planning and conduct of clinical trials across diseases and in LMICs.

## 2 Materials and equipment

As this method for community engagement is primarily conversational in nature, minimal materials are needed for its conduct. However, we’ve included an example of supplies used by this team of investigators for community consultation meetings and some of the recruitment and retention activities suggested by the community.

### 2.1 Sample materials for community consultation meetings (before and during trial)

- Quiet and private meeting space
- Chairs for participants
- Projector and screen, posters, or other visual aids
- Note-taking tools (computers, notepads, pens, etc.)
- Refreshments for participants

### 2.2 Sample materials for enhanced recruitment (as suggested by community)

- Quiet meeting space for group learning and discussion
- Private meeting space for individual consent
- Video, presentation, handouts for educational components
- Printed consents and knowledge checklists
- Audio recordings of consent on tablets with headphones

### 2.3 Sample materials for retention peer groups (as suggested by community)

- Games, activities and educational materials for participants and families at “peer groups”
- Refreshments for participants and families
- Pain mitigation and distraction tools for injections (Buzzy® devices, fidget toys, etc.) (15)

## 3 Methods

A key feature of the RRCU's community consultation approach for trials is its use of both upfront and on-going co-design. This enables investigators to develop community-informed protocols and trial designs while remaining flexible as circumstances change and their understanding of community needs and preferences evolves. The GOAL-family of studies is a premier example of an RRCU research project being designed with continuous community engagement as a foundational strategy. Our approach is described below.

### 3.1 Community engagement studios (pre-trial consultation)

During protocol development for the GOAL trial, we held four “community engagement studios” adapted from the Meharry-Vanderbilt Community Engaged Research Core framework, to help shape the trial's ethical and operational design (16–18). Rather than identifying unmet needs, the studios were intended to elicit community perceptions, concerns, and practical recommendations that could be directly integrated into study design. These sessions took place in Kampala, Uganda and included 4–9 volunteer community consultants each. RRCU staff worked with community leaders to recruit participants who represented the broader population and key stakeholder groups, including parents, teachers, school administrators, and community leaders. Participants verbally consented to participation and were informed that all contributions would remain anonymous outside the setting of the conversation. They received transportation reimbursement, a nominal stipend, and a snack and beverage for their participation.

The studios, which lasted 60–90 min, were conducted by three trained qualitative researchers in familiar community settings, including schools and churches. Each studio began with a presentation in Luganda (local language) by an RRCU research nurse. The presentation outlined RHD, the rationale for clinical research, and principles of randomization. The topics were presented in plain terms using examples from the local context, and the presentation was followed by a question-and-answer session to ensure shared understanding and open dialogue. The research nurse then moderated a follow-up discussion, using prompts co-developed by the investigators and the qualitative researchers conducting the sessions. These adaptable prompts addressed trial elements the research team expected might create implementation challenges, including approaches to communication, consent and randomization, appointment scheduling, prophylaxis adherence, and follow-up. Sessions were recorded, transcribed, translated to English, and inductively thematically analyzed by the moderator and the two other qualitative researchers in attendance. The research team debriefed immediately after each session to capture key insights and group dynamics.

### 3.2 Community advisory board (during-trial consultation)

Community Advisory Boards have become a central feature of the GOAL-family of studies and RRCU's standard for trial design. They are also a suggested oversight approach for studies involving humans as research subjects, according to the *Uganda National Council for Science and Technology* (19). Composed of local leaders from health, education, political, religious, and civil sectors, along with people with lived experience of RHD, the Boards help connect investigators with the communities the serve. Members are selected through consultative processes to ensure balanced district representation and continuity throughout a trial. They regularly attend participant events, review study materials, advise on cultural and ethical issues, and help identify emerging concerns. Their input directly influenced several adaptations for the GOAL and GOALIE trials, including expanded radio sensitization, refinement of messages sent home to parents, and the addition of clinical demonstrations for caregivers during enrollment. By serving as trusted community ambassadors, the Boards have helped dispel myths, reduce misinformation, and reinforce community ownership of the research.

## 4 Results: community-generated implementation tactics

### 4.1 Priorities for design from pre-trial community engagement studios

**Figure 1:**
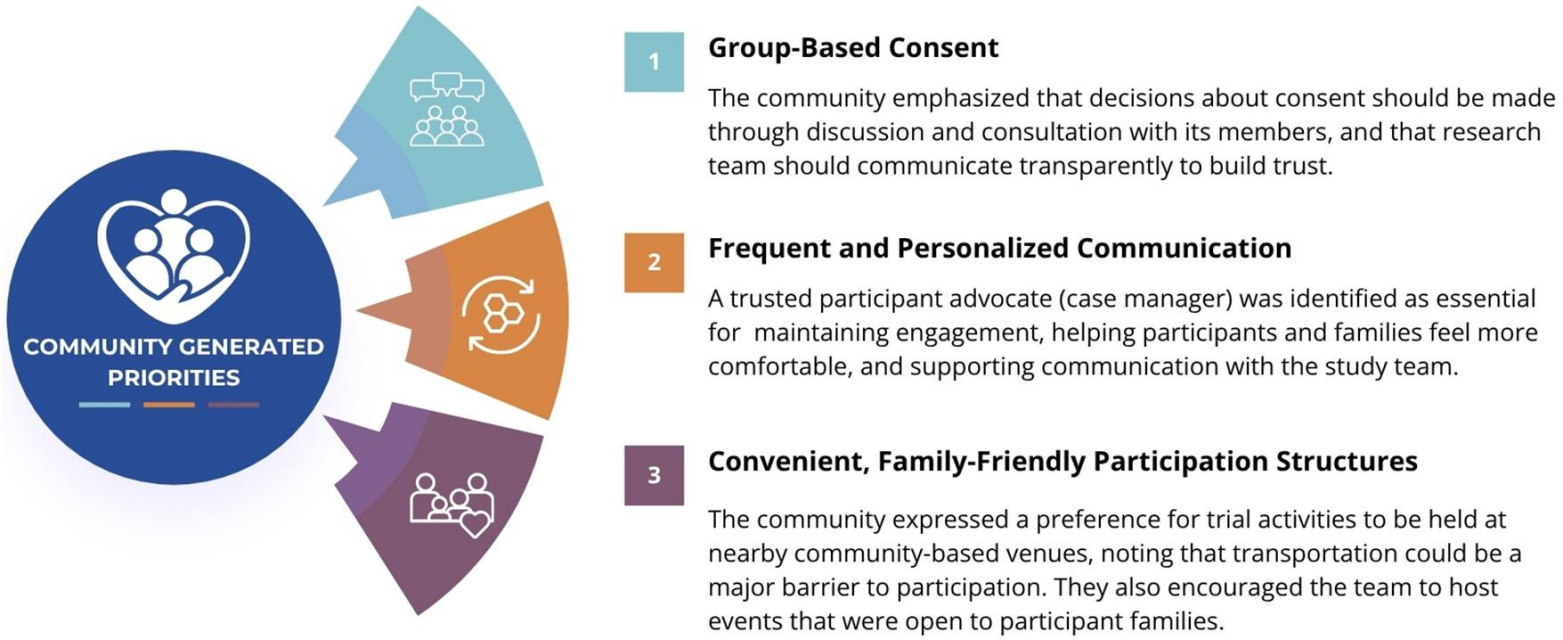
Community generated priorities for rheumatic heart disease trials in Northern Uganda.

Across the four community engagement studios held for the GOAL trial, community consultants identified barriers to screening, trial consent, participant follow-up, and medication adherence, while proposing culturally grounded solutions. Three priorities emerged (Figure 1). Families and stakeholders expressed strong preferences for: 1) interactive, group-based consent processes that clearly explained randomization and medications; 2) frequent and personalized communication with a trusted member of the research team throughout the duration of the trial; and 3) convenient, family-friendly participation structures for healthcare follow-up and medication delivery, ideally scheduled on weekends. Participants emphasized the importance of communal discussion, peer support, and geographically accessible engagement. These insights were translated into concrete design decisions throughout the GOAL trial, shaping recruitment, consent, engagement and retention, and adherence support for trial interventions. These tactics have subsequently been used by RRCU across the GOAL-family of studies and beyond.

#### 4.1.1 Priority 1: building understanding and creating opportunity for communal decision-making

The community engagement studios made it clear that a conventional consent approach—reviewing a written form prior to individual signature in private—would be insufficient to support understanding, trust, and enrollment. Community members emphasized the desire for education about RHD, the use of visual and narrative tools to explain randomization, and opportunities for communal discussion before individual decision-making.

**Figure 2:**
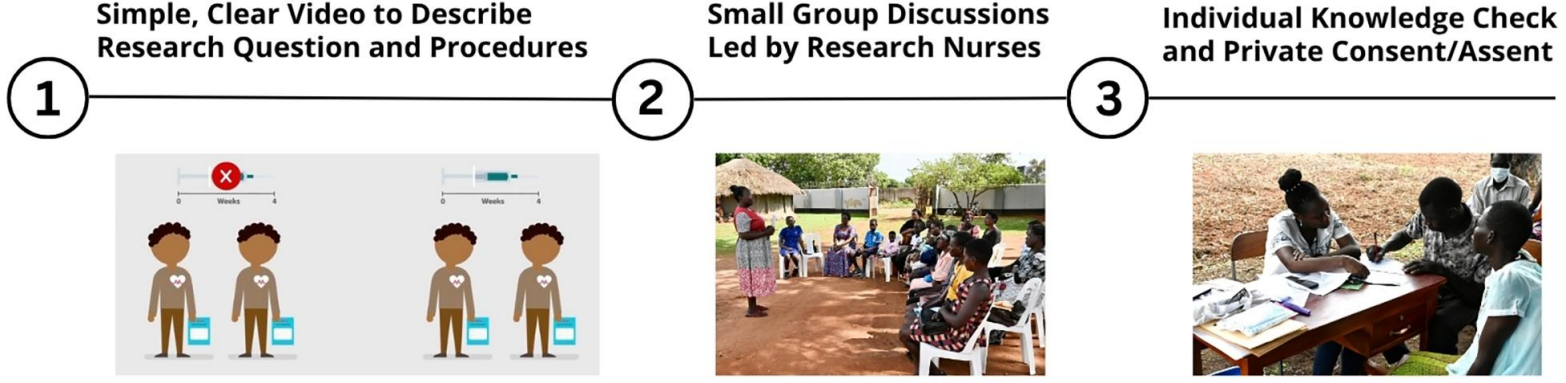
The RRCU approach to community-based consent.

In response, we co-developed a multi-step consent process that integrated regulatory requirements with community-informed practices (Figure 2). Following screening and determination of eligibility, small groups of 7–8 family units (children and their caregivers) moved through a structured consent preparation process designed to build understanding sequentially.

Consent at the GOAL trial office began with families viewing an animated video in the local language, which explained RHD, the trial's purpose, procedures, risks, benefits, and randomization (Figure 2). The video was iteratively refined based on community feedback, with familiar analogies—like a coin flip or soccer team assignment—used to explain randomization. The video was followed by a short, nurse-led educational session, using pictorial echocardiographic images to demonstrate normal cardiac anatomy and changes to the heart caused by RHD. This visual approach allowed families to contextualize RHD in concrete and relatable terms and positioned disease education as the foundation of informed decision-making.

Following the video, families participated in a small group discussion moderated by research nurses. This allowed caregivers to ask questions, share concerns, and hear perspectives from other families, which reflects the studio-identified, culturally important, norm of communal learning and decision-making. Common topics addressed questions around the safety of penicillin, the likelihood of disease progression or cure, implications for school attendance, and procedures for missed injection visits.

Before consent was obtained, study staff worked privately with each child-caregiver unit to assess comprehension using a brief, structured checklist. Misunderstandings were addressed through targeted re-education. Only after families demonstrated understanding of the study, were they approached for formal consent. All families who declined to enroll in the trial were referred to the local cardiology clinic for routine care and enrollment in the RHD registry.

The entire consent process took each child-caregiver unit an average of 90 min. This dynamic approach prioritized education, visual communication, and communal dialogue while maintaining individual autonomy and ensuring participants were sufficiently informed and empowered to make an intentional choice regarding participation. In the GOAL trial, more than 95% of eligible families consented to enrollment.

#### 4.1.2 Priority 2: improving communication and connection through a case manager strategy

Sustained engagement and long-term retention in the GOAL trial were achieved through a structured case manager model—directly informed by the community engagement studios—that provided personalized, longitudinal support to participating families. This relationship-focused strategy included structured communication, flexible delivery and child-centered care.

Once enrolled, each participant was assigned a dedicated case manager who served as the primary point of contact for approximately 100 children and their families. Case managers built long-term relationships with families throughout the study, monitoring treatment adherence, adverse events, peer group attendance, and overall wellbeing. They conducted routine bi-monthly phone check-ins to provide support and reminders for upcoming activities, with more frequent follow-up for children with lower prophylaxis adherence. When barriers to participation arose, case managers conducted home or school visits to support adherence, ensure continuity of care, and sustain engagement. Communication was continuous and bi-directional, with caregivers encouraged to contact case managers at any time with questions or concerns. These case manager and peer group systems (described below) addressed concerns raised during community engagement studios such as missed appointments, injection fear, and competing family obligations.

#### 4.1.3 Priority 3: engagement and retention using a family peer group and child-centered model for prophylaxis adherence

Community members emphasized the need for follow-up that was convenient, family-centered, and responsive to real-world barriers, with strong preferences for continuity and accessibility. In response, we assembled peer groups, each consisting of approximately 100 children from similar geographic areas, that met every four weeks, on Saturdays, at centrally located schools or community centers. Peer groups followed predictable, long-term schedules, allowing families to plan participation in advance and reinforcing the cadence of care.

**Figure 3:**
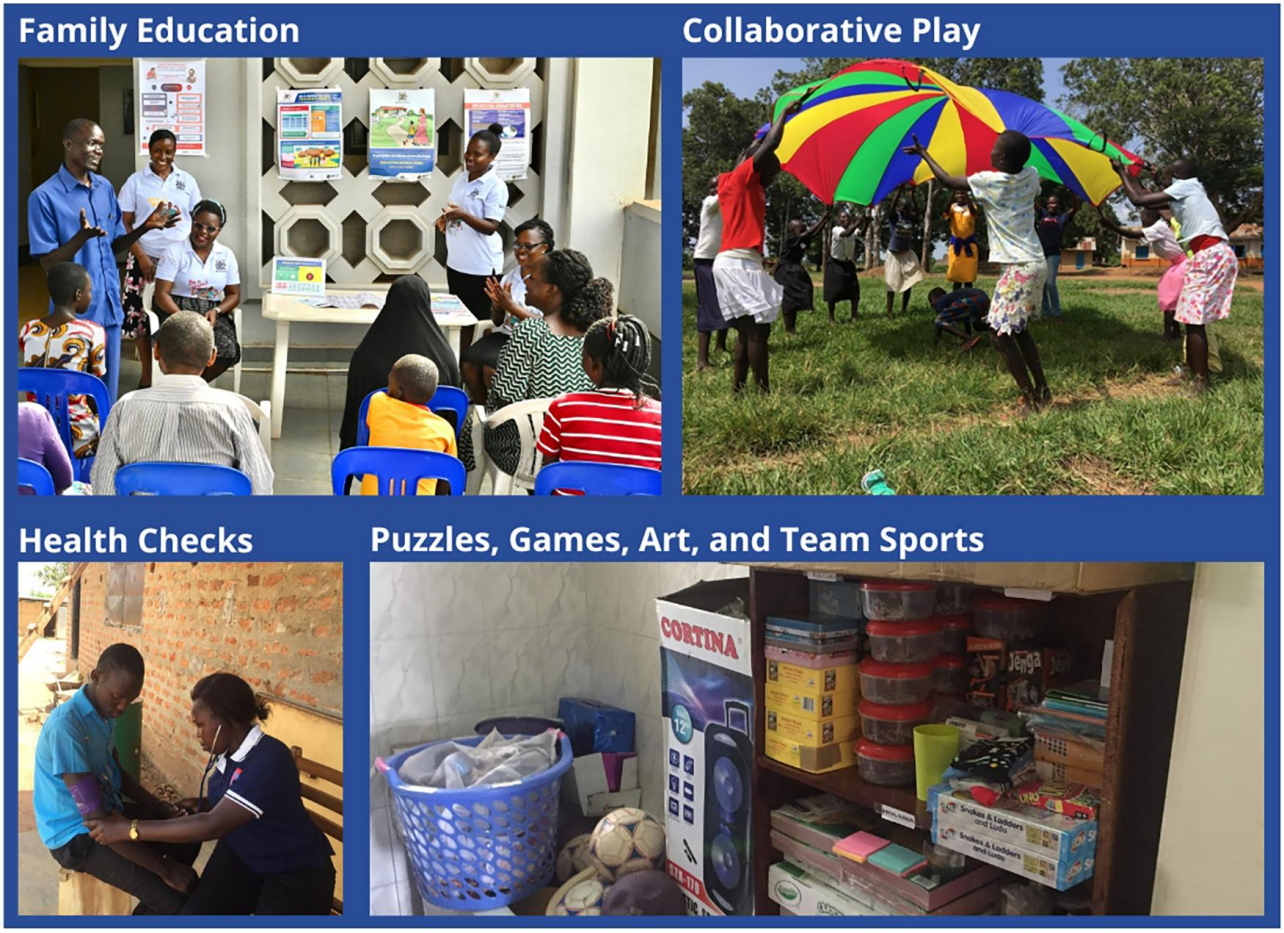
Peer group activities.

Children attended peer groups alongside a parent or guardian, creating parallel opportunities for social connection, shared learning, and peer support among caregivers. Based on feedback from the community engagement studio participants, peer group sessions were offered separately by study arm to reduce cross-arm stigma and concern. Integrated clinical review, data collection, and administration of prophylaxis (for the treatment study arm) were paired with structured educational and recreational activities (Figure 3). Education sessions addressed RHD, sore throat prevention, malaria, and adolescent health, while games, music, dance, art, and crafts fostered enjoyment and peer bonding. Skills-based activities, such as making liquid soap or reusable sanitary products for older girls, further increased the perceived value of participation for families. Beyond logistics, peer groups fostered a sense of social identity and belonging for both participants and caregivers.

Support to encourage attendance included individualized participant calendars and regular contact via SMS or calls, through which families were notified of upcoming peer group visits and received supportive communication to confirm attendance and emphasize the importance of timely prophylaxis. When attendance was an issue, case managers worked with families to adapt plans rather than allowing doses to be missed. For participants unable to attend peer groups, alternative delivery methods, including home, school or office visits, ensured continued prophylaxis.

Trained nurses administered monthly benzathine penicillin injections at peer group using standard pain-reduction techniques such as Buzzy® devices (15) and lignocaine. Children were also able to select their injection side, choose the nurse administering the injection, and use distraction techniques such as puzzles, guided imagery, conversation, or play-based activities. Nurses emphasized rapport-building, reassurance, and emotional support to reduce fear and anxiety.

These combined approaches reframed injectable prophylaxis as a predictable and supported experience. By combining structured reminders, outreach for missed visits, flexible delivery options, and child-centered pain and anxiety management, the GOAL trial sustained over 99% adherence to injectable secondary prophylaxis, delivering 10,284 of the 10,374 scheduled injections.

The GOALIE trial also introduced a tailored adherence support model for children randomized to oral penicillin prophylaxis. More than 500 children received a monthly supply of oral penicillin, with adherence supported through continuous, proactive engagement. Recognizing forgetfulness as the most common barrier, the program has emphasized layered reminders and social support, including patient and caregiver education, alarm clocks, weekly reminder phone calls, and personalized counseling. Children struggling with adherence were proactively identified and offered individual meetings to understand barriers and co-develop targeted strategies, while small prizes and recognition (certificates, pencils, crayons) were used to reinforce high adherence. Dedicated health workers provided individualized follow-up, with active involvement of families, teachers, and school nurses—particularly for children in boarding schools.

Together, the case manager, peer group, and personalized prophylaxis delivery systems provided a supportive, and socially meaningful follow-up experience for children and caregivers alike. Retention in the GOAL trial exceeded 97%, including during the COVID-19 pandemic, with withdrawals rare and largely attributable to relocation. This model illustrates how community-designed, family-centered engagement strategies can support participation in long-term clinical trials.

#### 4.1.4 Recruitment through community sensitization, a public health partnership, and trust

Though not explicitly discussed during the community engagement studios, GOAL trial recruitment was grounded on a longstanding partnership between the investigators’ research institution (The Uganda Heart Institute) and the Uganda Ministry of Health, which collaborated with the Uganda Ministry of Education to deliver echocardiography screening as a public health program. As part of this program, school-based echocardiographic screening for RHD is conducted in conjunction with routine outreach and service delivery, rather than as a research activity. The trial recruitment strategy was therefore designed to ethically and transparently transition families from public health screening to informed consideration of research participation.

Awareness efforts began before trial enrollment, emphasizing education over recruitment. Before school screening, students and teachers received education on RHD, its risk factors, and the screening process, including who the team was, why screening was being conducted, and what the results would mean. These well-informed students and teachers helped the research team spread the information in their communities, with children relaying details to parents and teachers providing clarification as needed. Screening results were provided on written forms with contact information, enabling parents to reference the information and connect with the clinical team.

Broader community awareness was reinforced through complementary channels. Educational radio talk shows were conducted with participation from community representatives alongside study team members to discuss RHD and the goals of screening and early intervention. In addition, individuals living with advanced RHD shared personal experiences, helping communities understand the consequences of untreated disease and the importance of early detection.

Trust-building was central to recruitment. Engagement of district health officers, district education officers, and community advisory board members ensured that local leadership was informed and visibly involved, which increased community confidence in both the screening activities and subsequent research. During school screening, teachers and learners were again engaged in discussions about RHD and the purpose of screening, reinforcing understanding and normalizing participation.

For families whose children had a positive screening echocardiogram and were invited to consider enrollment, recruitment emphasized transparency and shared understanding. When parents brought their children for enrollment visits, echocardiography was repeated and findings were explained in detail, allowing caregivers to directly visualize, discuss, and understand the diagnosis. This idea was brought forth through conversations between the investigators, community members and participant parents early in the GOAL trial launch. The echocardiography observation and consultation process enabled parents to make decisions from an informed and experiential standpoint rather than abstract explanation alone. This visual transparency strengthened understanding, trust, and confidence in clinical decision-making and consent, and has become a standard component of enrollment and engagement in RRCU trials.

Collectively, these strategies resulted in strong and timely enrollment. By embedding recruitment within a trusted public health framework and prioritizing education and transparency, the GOAL trial successfully moved families from awareness to informed commitment.

### 4.2 Ongoing input from community advisory boards and the broader community in response to challenges and misinformation

While most of the trial protocols were designed prior to project launch, the investigative team remained cognizant of and responsive to ongoing community concerns, needs and suggestions beyond the community engagement studios and throughout the duration of the studies. Here we describe additional considerations and adaptations RRCU employed during the launch and conduct of the GOAL and GOALIE trials as part of its flexible approach to community-based design and implementation.

#### 4.2.1 Continuous education and engagement

Despite strong early enrollment, the GOAL trial encountered challenges in some remote communities where limited awareness of RHD and the study contributed to rumors, stigma, and a temporary drop in participation. These challenges revealed that high-quality consent and engagement at the point of enrollment must be complemented by broader, ongoing district-level sensitization. In response, as a core element of study conduct during GOALIE, the research team implemented targeted outreach through meetings with community leaders, public radio programs, and facilitated community discussions in particular engaging people living with RHD in the community, locally referred to as “expert clients.”

#### 4.2.2 The importance of brand recognition

Per community recommendations, early and prominent co-branding with the Uganda Heart Institute—a nationally recognized and trusted provider of cardiovascular care—was intentionally prioritized to establish legitimacy and trust from the outset. Clearly identified study offices with visible GOAL/GOALIE and Uganda Heart Institute signage provided families with reliable, accessible points of contact beyond scheduled visits. Study staff wore GOAL/GOALIE uniforms and nametags, trial vans were clearly marked, and all materials were consistently branded, with key resources co-branded with Uganda Heart Institute logos to reinforce institutional credibility. Engagement with district health and education leaders, regular radio talk shows on RHD and sore throat management, and close collaboration with referral hospitals further embedded the trial within existing community, health, and education system structures.

#### 4.2.3 Compensation philosophy

RRCU, with community input, designed trial participant compensation to offset real participation costs without undue inducement. Rather than incentivizing enrollment or retention, our approach focused on preventing financial hardship for families, particularly related to transportation –a concern that was brought up multiple times during the community engagement studios. Participants or guardians received modest transport reimbursements at study visits (USD $5–15, based on distance) and a small appreciation payment (about USD $27) at trial completion. Compensation was locally determined and reviewed with ethics committees and stakeholders, reinforcing trust and voluntary participation.

#### 4.2.4 Expanding communication and sensitization infrastructure

GOAL showed that sustained, proactive communication with the wider community was key. GOALIE, expanded district radio programming about RHD, sore throat management, and the purpose of research, complementing peer group–level engagement, and reducing the spread of misinformation among non-participating groups. Communication with participants also became more systematic, using weekly SMS messages via UNICEF's “RapidPro” platform (20) for education, encouragement, and visit reminders, plus phone calls and home visits as needed. Furthermore, digital tracking helped flag missed visits, with 90% of cases resolved within one week. Following parent recommendations, GOALIE case managers also helped parents form “neighborhood teams” to boost family engagement and support. As part of this initiative, parents volunteered as team leads, reminding others about upcoming peer groups or trial activities during encounters at church, markets, or other community gatherings. Parents indicated that this program enhanced rapport and enthusiasm, while also offering an alternative communication channel for those who were less accessible by phone.

## 5 Discussion

The GOAL-family of trials demonstrate that rigorous, ethically sound clinical research is achievable in low-resource settings when communities are engaged as partners from the outset. By integrating community values into trial design, enrollment, follow-up, and communication strategies, this RRCU framework achieved exceptional recruitment, retention, and adherence—outcomes that exceed those of many traditional clinical trials and directly address long-standing concerns about feasibility in similar contexts.

Several features explain the success of this approach. First, community-informed co-design ensured that trial procedures aligned with daily realities, priorities, and expectations of families. This is supported by evidence from systematic reviews showing that community engagement interventions significantly improve recruitment and retention in clinical trials. Predictable, family-friendly peer group structures transformed follow-up visits from burdensome clinical encounters into socially meaningful events that combined care delivery, education, and connection. Such integration of health services with social support structures has proven effective across multiple disease contexts (21, 22); for instance, community-based adherence support interventions have demonstrated substantial improvements in pediatric HIV treatment outcomes in sub-Saharan Africa (23).

Second, continuous, two-way communication—through case managers, SMS, phone calls, and in-person outreach—enabled early identification of challenges and rapid, tailored responses, reinforcing trust and accountability. Mobile health interventions for medication adherence have shown consistent benefit in resource-limited settings, with meta-analyses demonstrating improved adherence across chronic conditions including cardiovascular disease (24). The multimodal communication strategy employed in GOAL mirrors successful approaches in tuberculosis treatment programs, where integrated SMS reminders, peer support, and case management have significantly reduced loss to follow-up (25).

Third, visible benefits to participants and communities, including health education, linkage to care, and respectful compensation practices, reinforced the perception of the trial as a shared investment rather than an extractive exercise. This reflects principles articulated in the Community-Engaged Research framework (26), where reciprocal relationships and tangible community benefits are essential for ethical research partnerships. Studies examining community perceptions of research participation consistently identify direct health benefits, respectful treatment, and ongoing communication as key factors influencing trust and willingness to participate (27–29).

The experience also highlights the importance of adaptability. Early challenges related to misinformation and stigma underscored that even well-designed trials must respond dynamically to community concerns. Research on stigma demonstrates that educational interventions, peer testimony, and transparent communication can effectively reduce misconceptions and improve care-seeking behavior (30–32).

The introduction of a Community Advisory Board, expanded radio sensitization, and enhanced transparency—such as echocardiographic demonstrations for parents— strengthened credibility and mitigated these challenges. Community Advisory Boards have been recognized as critical mechanisms for ethical oversight and cultural appropriateness in international health research, particularly in trials involving vulnerable populations (33–35). These refinements informed the design of the subsequent GOALIE trial, where engagement, adherence support, and communication strategies were further strengthened and tailored to oral prophylaxis regimens.

From a global perspective, the GOAL-family framework has important implications for RHD research and beyond. It challenges the assumption that high adherence and retention are unattainable in resource-constrained settings and provides a scalable model for conducting complex, longitudinal trials in pediatric populations. This counters persistent assumptions that have historically limited research investment in low-resource settings, as documented in analyses of global clinical trial distribution and participation disparities. The principles underpinning this framework—co-design, integration of care and education, structured social support, and sustained dialogue—are directly applicable to future RHD studies, including Group A Streptococcus vaccine trials. They also have implications for how to support people diagnosed in the public health system with RHD, even more important now that routine RHD screening using echocardiography has been endorsed by the 2024 World Health Organization guidelines (36).

Ultimately, the GOAL-family experience illustrates that scientific rigor and community partnership are not competing priorities but mutually reinforcing ones. This principle has been articulated in frameworks for community-engaged research across disciplines, emphasizing that authentic partnerships enhance both ethical integrity and scientific quality. Sustained success in prevention research will require moving beyond community participation toward genuine co-leadership, ensuring that the populations most affected by disease remain central to discovery, implementation, and long-term impact. Such transformation requires institutional commitment, equitable resource allocation, and recognition of community expertise as essential to the research enterprise—a shift increasingly advocated in global health research ethics and practice.

## Data Availability

The original contributions presented in the study are included in the article/Supplementary Material, further inquiries can be directed to the corresponding author.
