# Supplementary material for: Community-informed trial design and implementation to optimize recruitment and retention in low-income settings: lessons from rheumatic heart disease trials in Uganda
Source: Front Cardiovasc Med. 2026 Jul 13;13:1851733. doi: 10.3389/fcvm.2026.1851733 (PMC13402455; doi:10.3389/fcvm.2026.1851733)
Supplement: Supplementary file 1 [file Datasheet1.docx]

1. **Consent Knowledge Checklist for Enrollees (GOALIE)**

This tool was used near the conclusion of the consent process, after potential participants and their caregiver had watched the project consent video, participated in the group discussion with a research nurse, and either read or listened to the complete trial consent document. The checklist was administered by a staff member during an individual meeting with the child and caregiver pair. The staff member asked the potential participant and caregiver these study-related questions to ensure that they understood the main points of the trial and could make an informed decision about participation. If the child of caregiver did not know an answer or needed clarification, the staff member provided further explanation until everything was clear. The checklist starts with a practice question about posho, a local food, to confirm that participants understood how to respond to multiple-choice questions. The administration of this checklist helped ensure that trial participants were able to offer their true *informed* consent for the projects, a unique component of the family of studies.


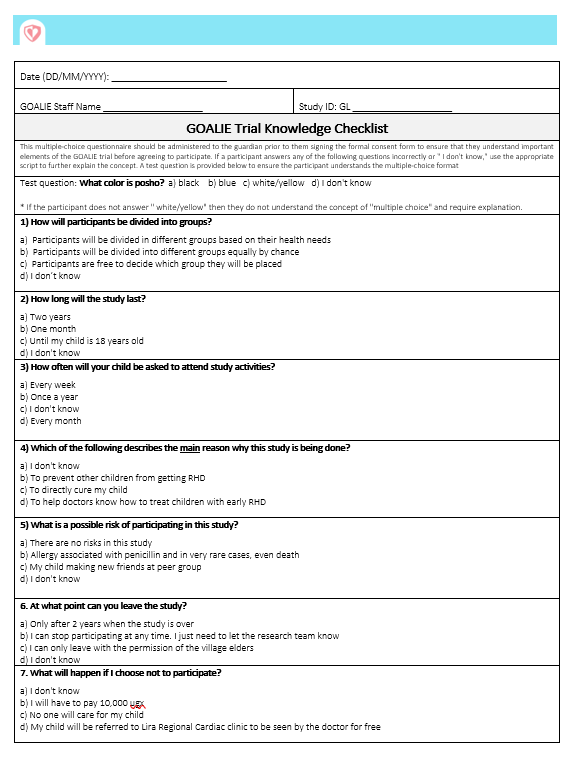


1. **Enrollment and BPG Tracking Card and Health Booklet (GOAL)**

To track important participant details and support workflow during peer groups sessions – where internet access was limited – project staff used physical cards with handwritten notes. The front of the card, which was completed at enrollment, recorded contact information and trial specifics such as participant ID, study arm, and assigned peer group. The second side of the card helped case managers document peer group attendance, prophylaxis adherence, and pain management preferences.

To monitor concomitant medication and illness, participants were also asked to maintain and share an individual health booklet or “passport” throughout the duration of the study. The booklet concept aligned with the community’s practice of tracking health information and doctor visits in notebooks. Complementing data collected during routine check-in calls with participants, this tool helped case managers understand participants’ health concerns and activities, a suggestion made by community members and required by trial protocols.

After each peer group, staff entered updated data from these materials into digital participant profiles on a secure online database. When not in use at peer group, the cards were securely stored in locked cabinets at the study office, and participants took their booklets home with them. The cards and booklets supported case managers in operationalizing peer groups, building individual relationships with participants, monitoring participants other medications and health, and customizing prophylaxis delivery plans – all of which were suggested by the community during the design studios.


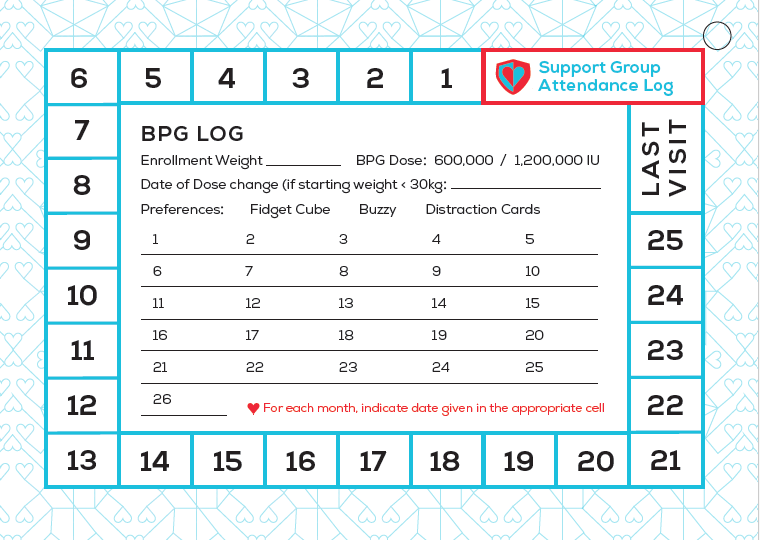

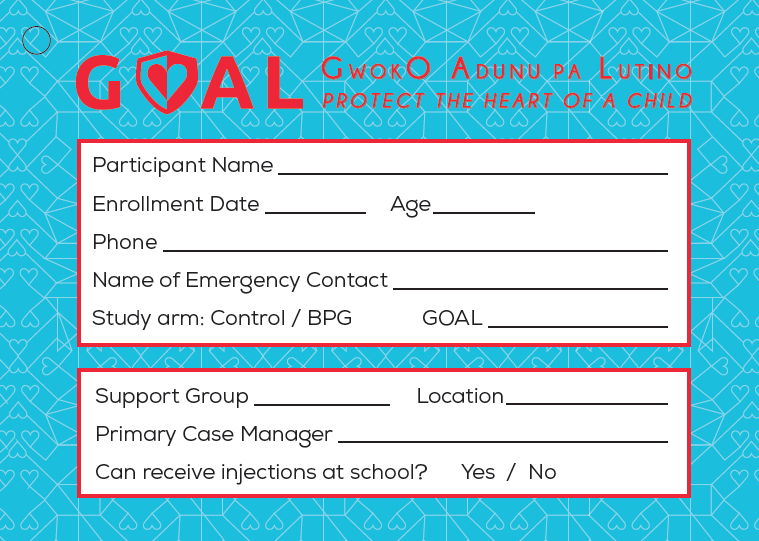

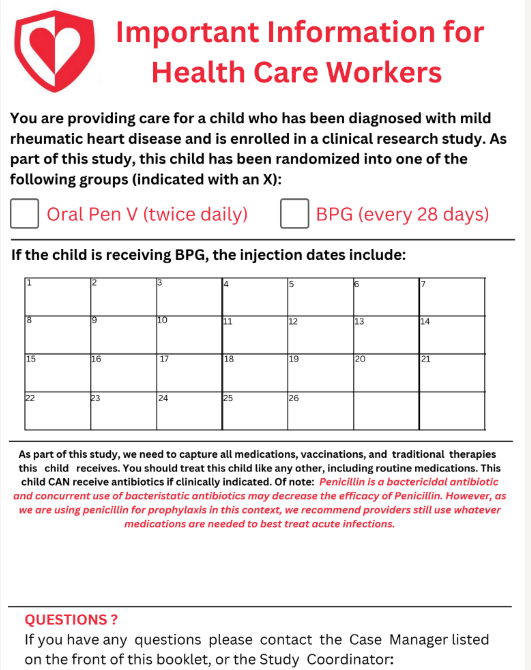

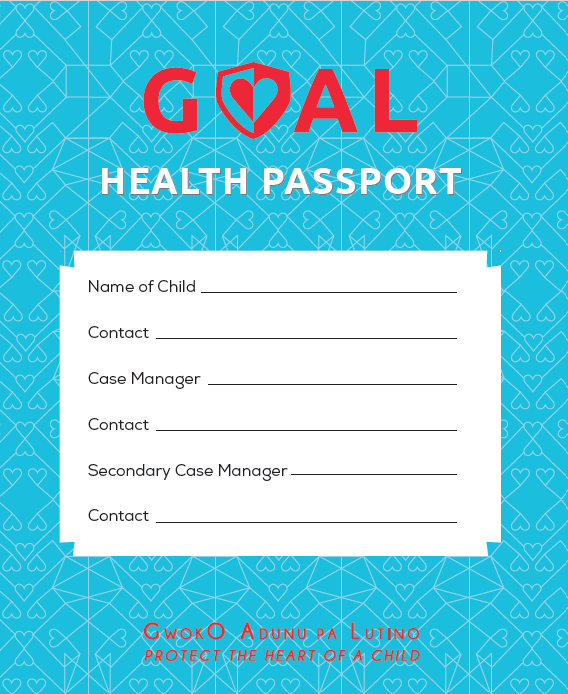


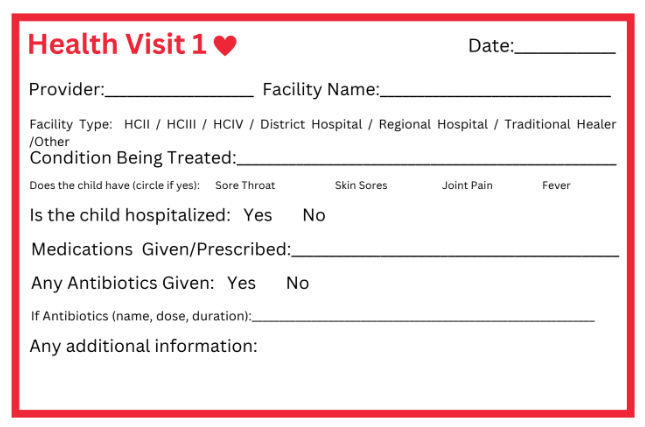


1. **Enrollment Flow Sheet (GOALIE)**

Project staff tracked participants’ progress through the enhanced consent process using a paper Enrollment Flow Sheet, which were carried by each child-caregiver pair through the study office during their visit. The process was divided into stations, and each participant received colored stickers as they completed each step, allowing team members to easily monitor their status. The forms were checked when attendees signed out of the office, ensuring complete enrollment and helping instill confidence in the program by displaying the project’s polished and organized processes.


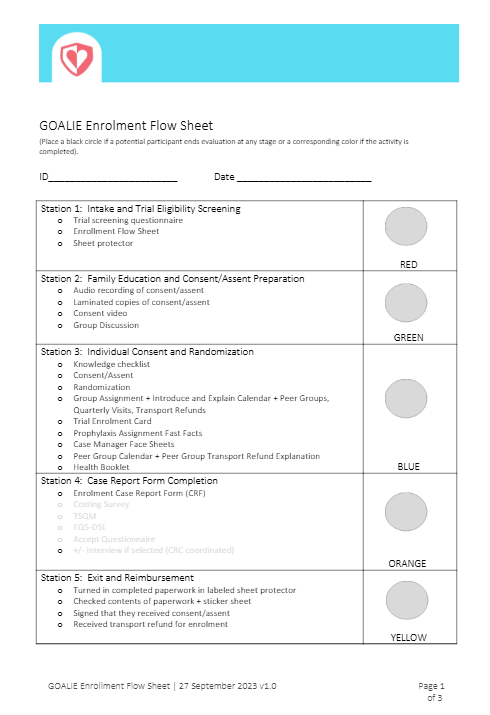


1. **Branded Materials (GOAL and GOALIE)**


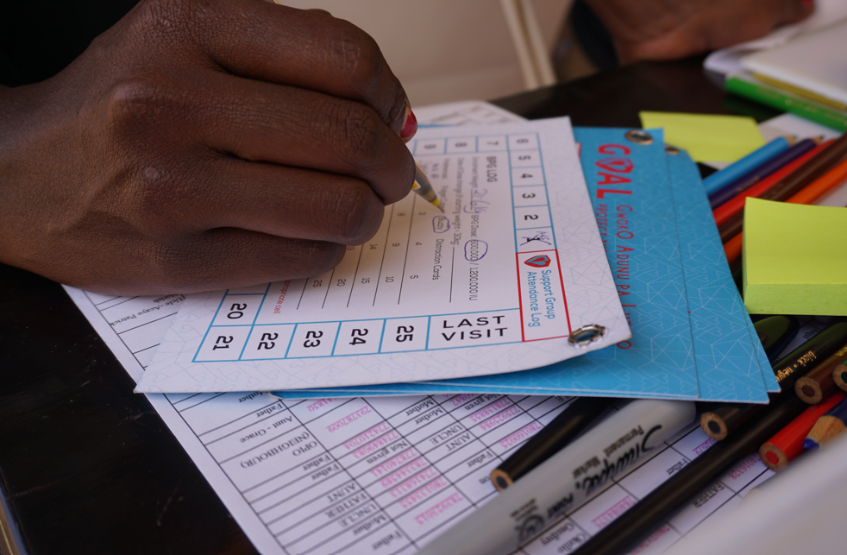

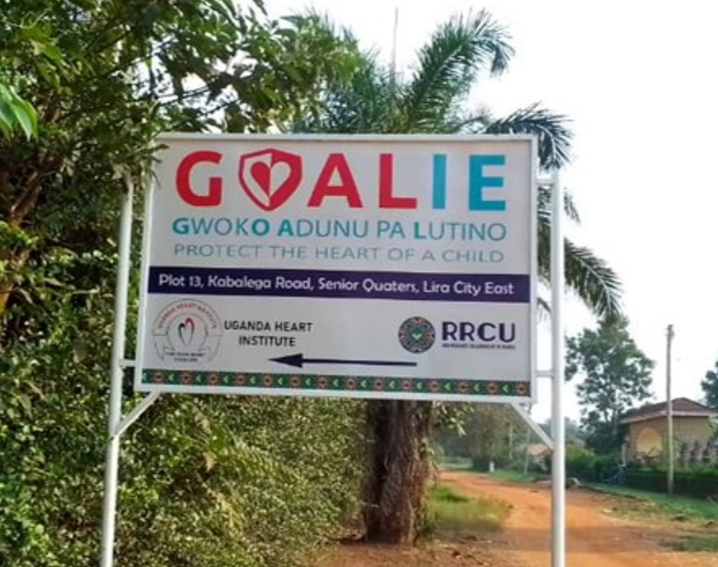
Given the necessary integration of the GOAL family of projects within local communities, a cohesive and professional image was essential for building trust. The projects consistently used project logos and branding (materials, signs, marked vehicles, uniforms, etc.) to promote uniformity across the program and support brand recognition. To further build confidence in the projects, the logos were frequently displayed alongside that of the Uganda Heart Institute (the organization overseeing the project), which is well-regarded in the community.
